# CFD simulation of silica dispersion/natural rubber latex mixing for high silica content rubber composite production

**DOI:** 10.1039/d4ra01348d

**Published:** 2024-04-18

**Authors:** Ekaroek Phumnok, Phonsan Saetiao, Panut Bumphenkiattikul, Sukrittira Rattanawilai, Parinya Khongprom

**Affiliations:** a Department of Chemical Engineering, Faculty of Engineering, Prince of Songkla University Songkhla 90110 Thailand parinya.kh@psu.ac.th; b Industrial Technology Department, Petroleum Technology Program, Faculty of Industrial Education and Technology, Rajamangala University of Technology Srivijaya Muang Songkhla 90000 Thailand; c Simulation Technology, Digital Manufacturing, Chemicals Business, SCG 1 Siam Cement Road, Bang sue Bangkok 10800 Thailand; d Department of Chemical Engineering, Faculty of Engineering, The Thai Institute of Chemical Engineering and Applied Chemistry, Chulalongkorn University Bangkok 10330 Thailand; e Air Pollution and Health Effect Research Center, Prince of Songkla University Songkhla 90110 Thailand

## Abstract

High silica contents rubber composites are favored in the green tire industry for their ability to reduce rolling resistance. However, achieving effective silica dispersion in natural rubber, particularly at high silica content, poses a challenge. In addition, the choice of impeller configuration significantly influences mixing performance, especially in commercial production, which requires large mixing tanks. Therefore, understanding the scaling-up process for this mixing system is essential. This research aims to investigate the mixing of silica dispersion in natural latex, specifically focusing on a high silica content regime. The flow characteristics of each liquid phase were simulated by employing the Computational Fluid Dynamics (CFD) approach, with a two-fluid model serving as the model based. Analyses were conducted on two variants of stirred tank reactors including four baffles and flat bottoms. Four configurations of Rushton turbine impellers were considered: four 90° blades (RT4-90), four 45° blades (RT4-45), six 90° blades (RT6-90), and six 45° blades (RT6-45). The simulations revealed that the 90° blade promoted the radial flow, while the 45° blades enhanced axial flow, through the process of diverting a significant proportion of the fluid above impeller, this regime effectively increases the liquid's velocity. Increasing the number of blades led to a more homogeneous velocity profile within the impeller region. Additionally, higher fluid velocity was observed in a larger mixing tank. In a smaller tank, the impact of impeller design (number and angle of the impeller) on mixing time was less pronounced. However, the mixing time decreased with the increasing blade number in a larger tank. In addition, the 45° blade angle tends to decrease the mixing time. The optimum design is the Ruston turbine with six blades set at a 45° angle. Furthermore, the upscaling criteria that were proposed by Norwood and Metzner were used into this inquiry. The suggested scaling criterion was consistently applied to the mixing of high silica natural latex, with no deviation exceeding 10%.

## Abstract 2

The impeller configuration significantly influences on the hydrodynamics and mixing performance. The optimum design is the Ruston turbine with six blades set at 45 angle. The Norwood and Metzner scaling criteria is reliable to scale up of natural latex containing high silica content stirred tank.

## Graphical abstract

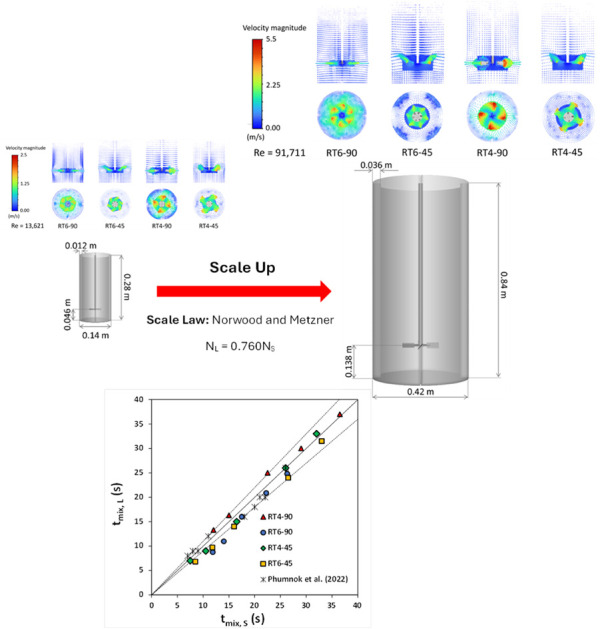

## 1. Introduction

Natural rubber composites, recognized for their unique mechanical properties, have emerged as crucial materials in various industries.^1–3^ Fillers are essential components in natural rubber composites, developed to enhance the quality of composite products. The tire industry is increasingly incorporating silica (SiO_2_) filler as an additive. The utilization of silica fillers in rubber compounds has been demonstrated to notably decrease the rolling resistance of tires. This phenomenon is attributed to silica's elevated specific surface area and its capacity to interact with the rubber matrix, thereby augmenting the reinforcing effects. Consequently, the incorporation of silica-reinforced rubber offers technical benefits over carbon black-filled rubber, notably in terms of reduced rolling resistance. This reduction enhances not only fuel efficiency but also results in decreased carbon dioxide emissions, rendering silica-filled rubber compounds an increasingly appealing option for tyre manufacturers striving to comply with rigorous environmental standards while enhancing overall vehicle performance. Furthermore, silica continues to induce low hysteresis, high hardness, and robust abrasion resistance.^1,2,4,5^

Additionally, silica can be created as nanofillers, with the ability to alter its surface characteristics from hydrophilic to hydrophobic. This flexibility broadens its potential applications, encompassing the utilization of nanocomposite silica/recycled NBR rubber and the facilitation of superhydrophobic attributes.^6–8^ Furthermore, this characteristic lends itself to developing nano-silica epoxy adhesive, which enhances the adhesion between steel and concrete.^9^ Rubber composites can be produced using a dry process and a wet process. Dry mixing, a traditional procedure that occurs before vulcanization, entails the mechanical mixing of filler particles with dry rubber in a direct manner. Drawbacks of this method include non-uniform distribution of the filler and high-power consumption.^10,11^ The wet process has recently emerged as a solution to these challenges, enhancing filler distribution and mechanical properties of vulcanized rubber. It involves dispersing filler particles in a liquid medium, such as water or solvent, before mixing with other components. This method facilitates more uniform filler distribution by preventing agglomeration and promoting better interaction among components. The liquid medium's coating of filler particles reduces agglomeration and enhances their flowability, promoting homogeneous dispersion. In contrast, dry mixing, lacking these benefits, struggles to achieve uniform distribution due to increased agglomeration and inefficient dispersion. Furthermore, wet mixing consumes less energy than dry mixing due to reduced frictional forces, improved flowability, lower shear stress, and prevention of agglomeration. These advantages have positioned the wet process as a preferred method for silica-rubber compounding, streamlining mixing steps and lowering energy consumption in the rubber production process.^3,12–14^ Nevertheless, the natural rubber molecule and the silica filler are incompatible. Consequently, a number of techniques have been developed to increase the dispersion of silica in the rubber matrix. These methods include the use of coupling agents,^15–17^ the addition of a second filler,^11,18,19^ and the improved interface structure.^20,21^

The stirred tank is a key equipment for the wet process, with flow behavior and mixing in the system directly influencing product quality. However, experimental investigation of hydrodynamics in the mixing tank presents a formidable obstacle due to the complex characteristics of flow behavior and the intense interaction that defines multiphase flow. Consequently, the utilization of Computational Fluid Dynamics (CFD) has become increasingly prevalent in the investigation of fluid mixing and flow processes within stirred tanks. Researchers utilized CFD methodologies to examine flow patterns in vessels with high aspect ratios featuring multiple impellers. They have also assessed the mixing performance of various impeller designs across different fluid conditions.^22–25^ This approach has provided valuable insights into the behavior of diverse impeller types, contributing to the enhancement of more efficient mixing processes. In industrial applications, the study of flow characteristics of continuous and dispersed phases of highly viscous liquid mixing using CFD has become a critical field of research.^26,27^ In such systems, the viscosity disparity between the continuous and dispersed phases significantly impacts the mixing process. CFD serves as a powerful tool for analyzing flow behavior,^28,29^ understanding complex interactions between phases,^30,31^ and optimizing mixing performance.^32–34^ Engineers can optimize impeller geometry, rotational speed, and baffling arrangements based on CFD results to achieve improved mixing performance. In addition, the impact of different process factors, including impeller clearance, size, and liquid characteristics, on flow behavior and mixing performance may be investigated with the use of these simulations.^35–39^

Scaling the appropriate volume of a stirred tank can pose challenges, particularly when dealing with high-viscosity liquids. This issue arises due to notable changes in flow characteristics observed on the reactor scale. Various criteria for size reduction in stirred tanks have been suggested, considering their similarity in geometry, dynamics, and kinetics.^40^ Input power per mass, geometric identity, consistent average turnover time, and constant power input normalized by mass are the four criteria that are often considered for determining size. Geometric similarity is a critical aspect in scholarly discussions, where the speed of the propeller tip and analogous geometry remain constant. The choice of scaling criteria depends on the specific objectives of the study. The level of uniformity is a critical factor in assessing the efficiency of wet mixing procedure. Therefore, mixing time emerges as a suitable parameter for up-scaling stirred tank for this application.

The purpose of this research is to ascertain the hydrodynamic and mixing efficiency of a wet mixing process utilizing natural rubber and silica through the utilization of computational fluid dynamics (CFD) simulations. The core focus of the investigation was to assess the influence of impeller design and the scalability of stirred tanks.

## 2. Methodology

### 2.1 Reactor geometry

**Fig. 1:**
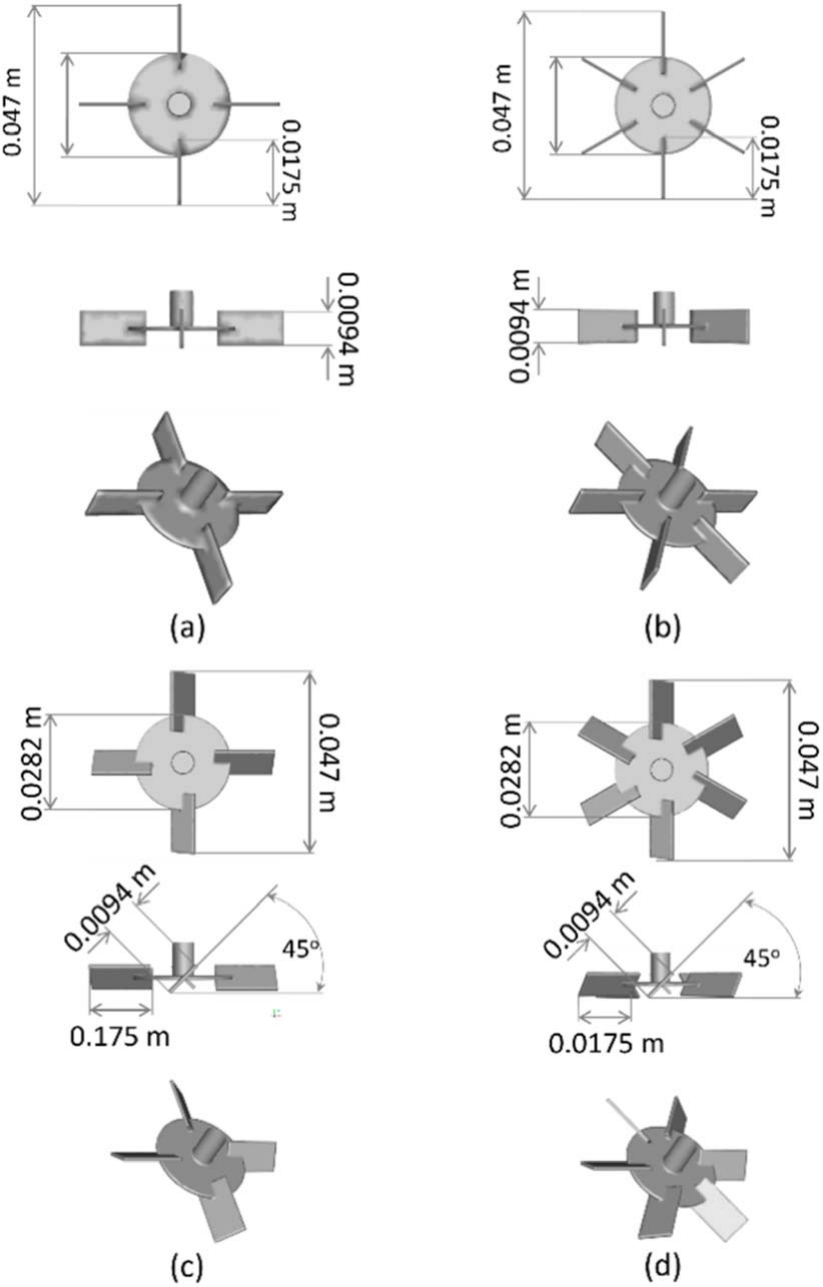
Configurations of the four-blades, 90° (RT4-90) (a) six-blades, 90° (RT6-90) (b) four-blades, 45° (RT4-45) (c) and six-blades, 45° (RT6-45) (d) Ruston turbine agitators.

In this study, a stirred tank with a flat bottom and four baffles was employed, as illustrated in Fig. 1. A variety of Rushton turbine impeller designs were implemented, comprising six 45° blades (RT6-45), six 90° blades (RT6-90), four 45° blades (RT4-45) and four 90° blades (RT4-90). As indicated in Fig. 1, the width and length of each blade are 0.0094 m and 0.0175 m, respectively. The stirred tank's configuration and installation are described in Table 1.^41^ The small, stirred tank has the following dimensions: 0.14 m in diameter and 0.28 m in height. The dimensions of the enlarged stirred tank were three times the dimension of the smaller tank. The correlations among the reactors were ascertained by employing eqn (1).1
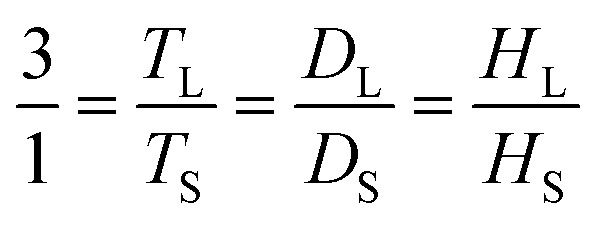


**Table 1:** Configuration and installation of the stirred tank

*H*/*T*	*C*/*T*	*D*/*T*
1	1/3	1/3

**Fig. 2:**
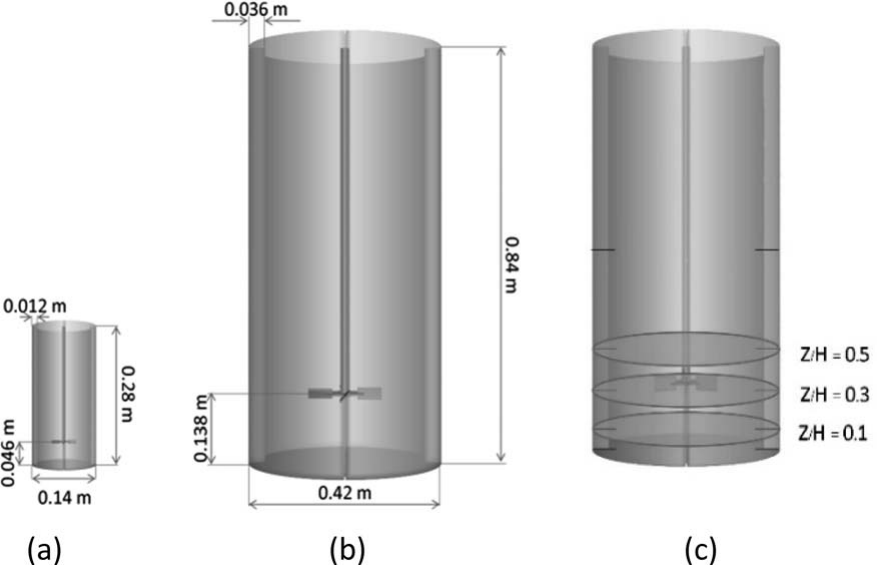
Small (a) and large (b) reactor tank configurations and (c) monitored position.

Fig. 2 illustrated the arrangement of the small and large stirred tanks. This study focused on the mixing of high-viscosity liquid. A 15% silica dispersion, serving as the high-viscosity fluid, was dispersed in natural latex, acting as the low-viscosity component. The densities of the 15% silica dispersion and natural latex are 1.132 g cm^−3^ and 0.9833 g cm^−3^, respectively. Natural latex and a 15% silica dispersion have respective viscosities of 12.52 mPa s and 140 mPa s.

### 2.2 Mathematical model

A two-fluid model using the Eulerian–Eulerian method was used in the current study in predicting the flow properties within a stirred tank. The interphase momentum transfer is influenced by several forces, such as drag, additional mass, lift, and Basset forces.^42^

However, this investigation focused solely on the drag force, as the effect of additional forces on the interphase interactions was found to be negligible.^43^ The *k*–*ε* turbulence model was utilized to predict the turbulent flow.^44^ The standard *k*–*ε* two equation turbulent model provides reasonable accuracy, time economy, and robustness for a wide range of turbulent flows.^45,46^ This model is formulated under the assumption that the Reynolds stress is proportional to the mean velocity gradient.^47,48^ The governing equations are stated as follows:

The continuity equation is2
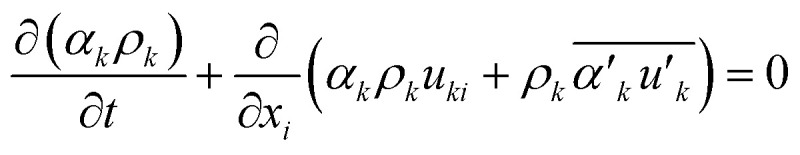


The momentum equation for phase *k* is given by3
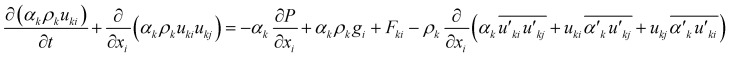


The Reynolds stresses, 
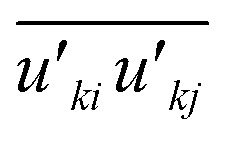
, is defined as4
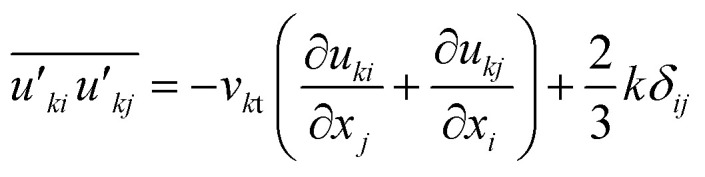


The term 
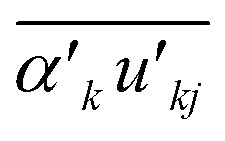
 is defined as5
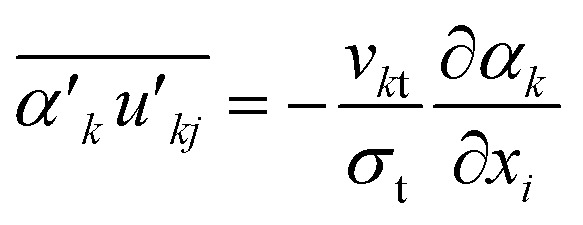


In eqn (3), term *F*_*ki*_, denotes the momentum exchange between phases. The drag force between phases can be predicted using eqn (6).6
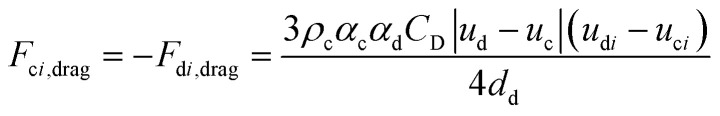


Schiller–Naumann drag model is often used in liquid–liquid systems, especially when the dispersed phase consists of droplets. It is simple and widely applicable. Thus, this drag model was applied in this investigation.7
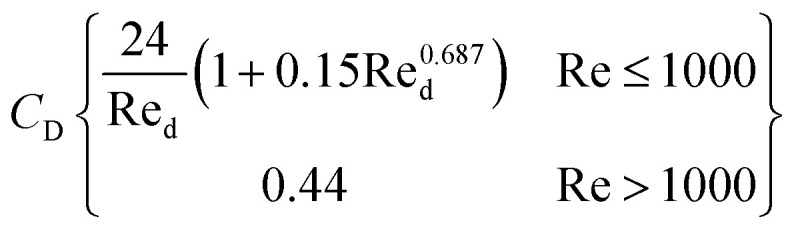
where8
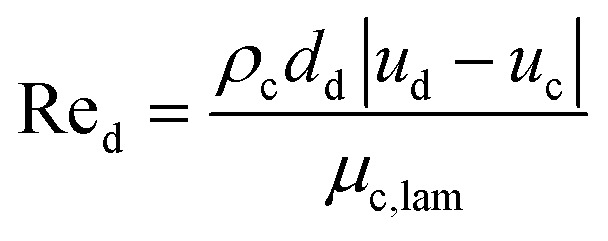
and9
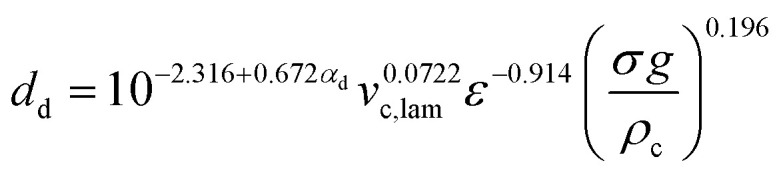


The standard *k*–*ε* two equation turbulent model was derived in the form10
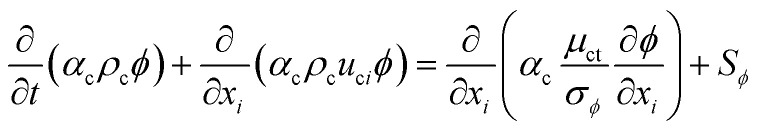


The details of the mathematical model can be found in our previous work.^29^

### 2.3 Numerical solution

**Fig. 3:**
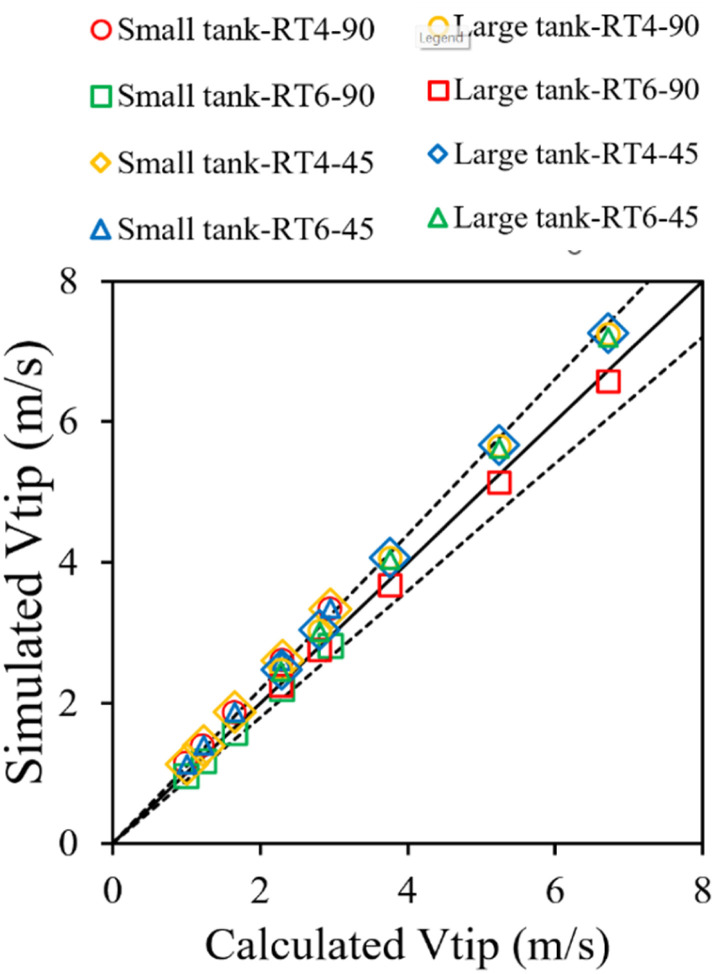
Comparison of tip velocity obtained from simulation and calculation.

The hydrodynamic flow properties of two immiscible liquids in a three-dimensional stirred tank were examined in this work. The flow behavior was modeled and simulated using Ansys-Fluent, a commercial program. The numerical solution of the governing equations was established using the finite volume approach. The gradients were computed according to the least-squares cell-based method. The discretization of turbulent kinetic energy, turbulent dissipation rate, momentum, and volume fraction was performed using first-order upwind approaches. The semi-implicit pressure-linked equation (SIMPLE) technique was utilized to establish a correlation between pressure and velocity within the momentum equation. This study considered multiple reference frames that have been extensively employed in previous studies.^42,43^ The tank, impeller, and baffle were subject to no-slip conditions at their respective wall boundary conditions. The velocity of agitation exhibited variability in both the impeller and rotor regions. Based on the grid sensitivity study conducted in our previous work,^29^ an optimum grid of 94 961 grids was utilized in this study. The numerical method and model parameters employed in this study were consistent with those established in our previous work.^29^ Simulation results based on those parameters were successfully validated against experimental data. Further validation of these parameters was conducted by comparing the tip velocity as shown in Fig. 3. A strong agreement between simulation and calculation results was observed.

## 3. Results and discussion

### 3.1 Flow behavior

**Fig. 4:**
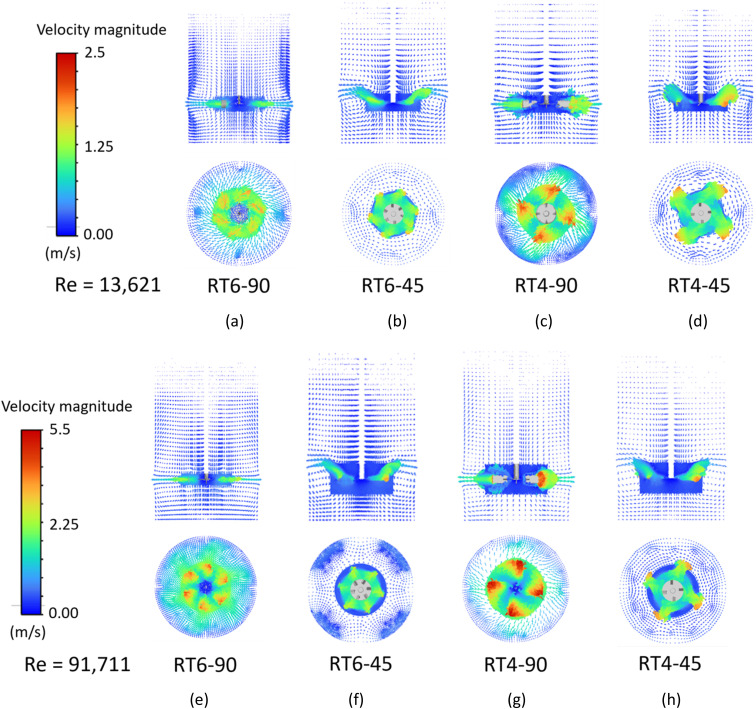
The velocity vector plot of a dispersed phase in small (a–d) and large (e–h) stirred tanks equipped with a Ruston turbine with 90° 6 blades, 45° 6 blades, 90° 4 blades and 45° 4 blades.

The velocity vector plot of the dispersed phase in stirred tanks with various impeller designs was shown in Fig. 4. For the 90° blade (Fig. 4a, c, e, and g), the fluid exhibits swift motion originating from the agitator's tip, moving radially, perpendicular to the shaft axis. Consequently, when reaching the boundary of the tank, the fluid undergoes separation, resulting in the formation of two different zones. One constituent of the substance rises and forms a circular path above the agitator blade, whereas another descends and re-enters below it. This radial flow pattern commonly observed in research with Rushton turbine agitators.^39^ Modifying the blade angle to 45° (Fig. 4b, d, f, and h) enhances the axial movement of the dispersed phase. Aligning the velocity vector direction with the blade angle improves axial flow, a phenomenon observed by several researchers.^49,50^ Due to the axial flow pattern, there is a notable reduction in the homogeneity of the velocity vector at the cross-sectional perspective at the *ZX* plane location of the agitator blade. When examining the impact of blade number, it was observed that an increased number of blades leads to a more homogeneous velocity profile inside the stirred tank because of the increasing of fluid–fluid interaction resulting in more turbulence and fluid circulation. The presence of a high-velocity zone is observed in close proximity to the blade tip, namely in the case of the four-blade configuration.

**Fig. 5:**
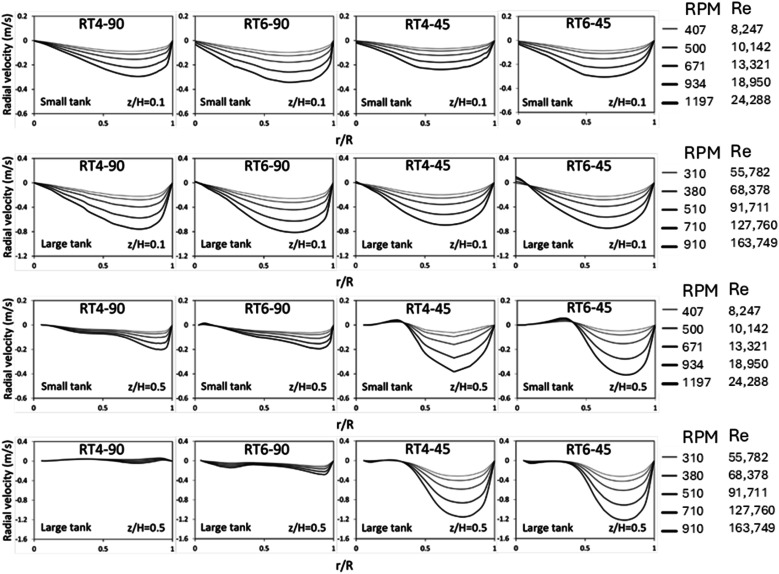
The influence of the Reynolds number on the radial distribution of the radial velocity of dispersed phase inside small and large reactors equipped with a Ruston turbine with 90° 4 blades, 90° 6 blades, 45° 4 blades, and 45° 6 blades at *z*/*H* = 0.1 and 0.5 above the tank bottom.

The radial velocity distribution during the dispersion phase for both small as well as large vessels was seen in Fig. 5. The measurements were performed at two distinct elevations, specifically, *z*/*H* equals 0.1 and 0.5, representing locations situated beneath and above the impeller's circumference, respectively, as shown in Fig. 2c. A velocity with a positive value indicates the movement of fluid through the agitator towards the wall of the vessel, whereas a velocity with a negative value indicates the flow of fluid from the wall towards the agitator itself. As the Reynolds number decreases (low rotational speed), the radial velocity demonstrates a homogeneous distribution. The observation of negative radial velocities in stirred tanks of varying sizes, when *z*/*H* equals 0.1, indicates the occurrence of fluid backflow from the tank periphery into the central region.^51,52^ At *z*/*H* = 0.5, the primary flow of fluid exhibits a counter-current motion, moving from the tank wall towards the tank center. The number of blades insignificantly impacts the radial velocity distribution, while the blade angle significantly influences distribution, especially at *z*/*H* = 0.5. For a 45° blade, a large amount of fluid axially flow upward, resulting in high radial velocity at this position. Moreover, it can be observed that the large tank exhibits a greater radial velocity in comparison to the small tank. Although the large tank operated at a lower rotational speed, its larger diameter resulted in a higher linear velocity. Therefore, the radial velocity of the large tank is higher.

**Fig. 6:**
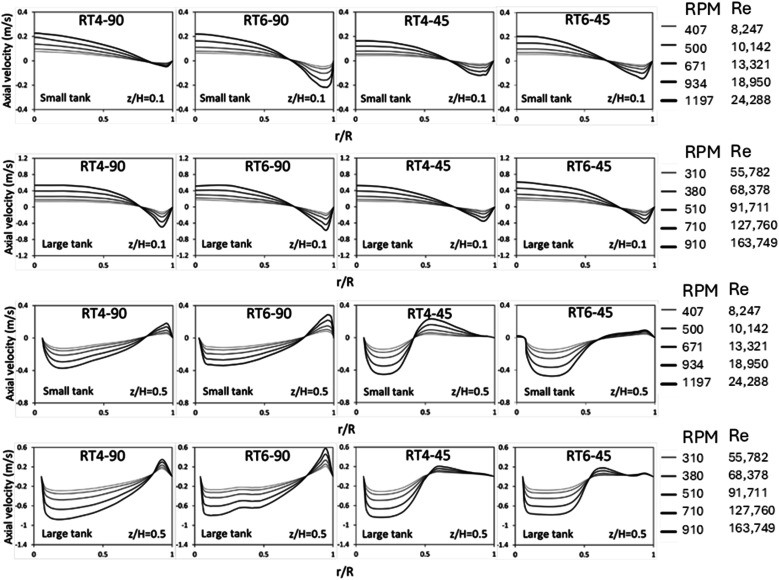
The influence of the Reynolds number on the radial distribution of the axial velocity of dispersed phase inside small and large reactors equipped with a Ruston turbine with 90° 4 blades, 90° 6 blades, 45° 4 blades, and 45° 6 blades at *z*/*H* = 0.1 and 0.5 above the tank bottom.

Fig. 6 illustrates the radial distribution of axial velocity in both small and large stirred tanks. The data shown demonstrates axial velocity variations at different Reynolds numbers. In fluid dynamics, positive magnitudes are indicative of upstream flows, while negative magnitudes correspond to downstream flow, in the context being discussed. At *z*/*H* = 0.1, the fluid flow exhibits two discernible regions. In the center zone, there is an upward flow, while in the annular region, downward flow is observed.

The flow pattern illustrates the phenomenon of fluid recirculation, wherein fluid originating from the annular region is redirected into the central portion of the tank. At a normalized height of *z*/*H* = 0.5, there is a discernible counterflow pattern observed, characterized by an upward flow within the annular area and a downward flow within the central region. This suggests the presence of fluid recirculation in the top region, consistent with observations in a previous experimental study.^38^ The impact of the blade number on the axial velocity distribution is minimal, with the axial velocity showing a slight increases with an increasing number of blade. However, the blade angle significantly impacts the radial distribution of axial velocity. For 45° blade, a large amount of fluid flows upward, extending the region of upward flow from *r*/*R* = 0.85–1.0 (90° blade) to 0.5–1.0 (45° blade). In addition, it can be observed that the large tank exhibits a greater axial velocity in comparison to the small tank because of larger linear velocity.

**Fig. 7:**
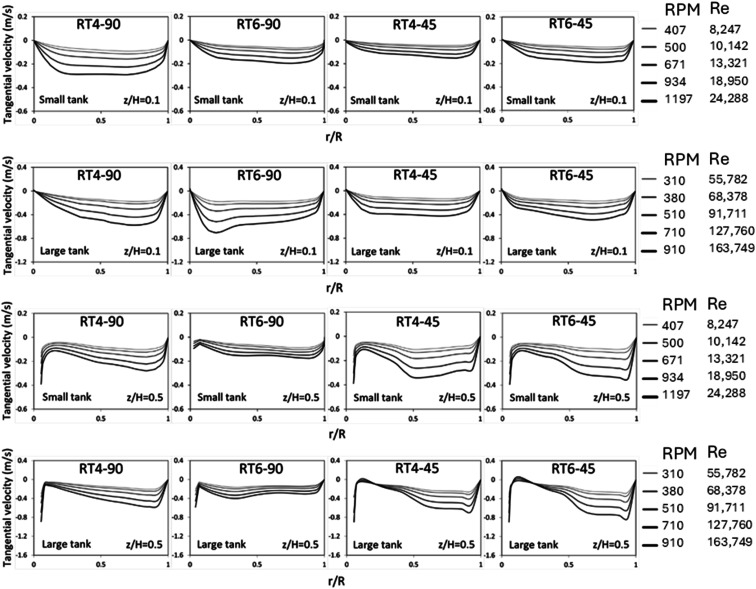
The influence of the Reynolds number on the radial distribution of the tangential velocity of dispersed phase inside small and large reactors equipped with a Ruston turbine with 90° 4 blades, 90° 6 blades, 45° 4 blades, and 45° 6 blades at *z*/*H* = 0.1 and 0.5 above the tank bottom.

Fig. 7 displays the radial distribution of tangential velocity. A positive velocity is indicative of fluid flow in a clockwise direction, whereas a negative velocity indicates a reverse flow. The tangential velocity of the 45° blade was seen to decrease at a certain axial location of *z*/*H* = 0.1. This reduction in velocity may be attributed to a decrease in the downward fluid flow inside the bottom region of the impeller. The tangential velocity distribution for the 90° blade exhibited a non-uniform pattern inside the large tank.^29^ However, at *z*/*H* = 0.5, high tangential velocity was observed for the 45° blade due to a large portion of fluid flowing upward of the impeller. Additionally, a large tangential velocity was observed in the large tank because of higher linear velocity.

### 3.2 Tip velocity and mixing time

**Fig. 8:**
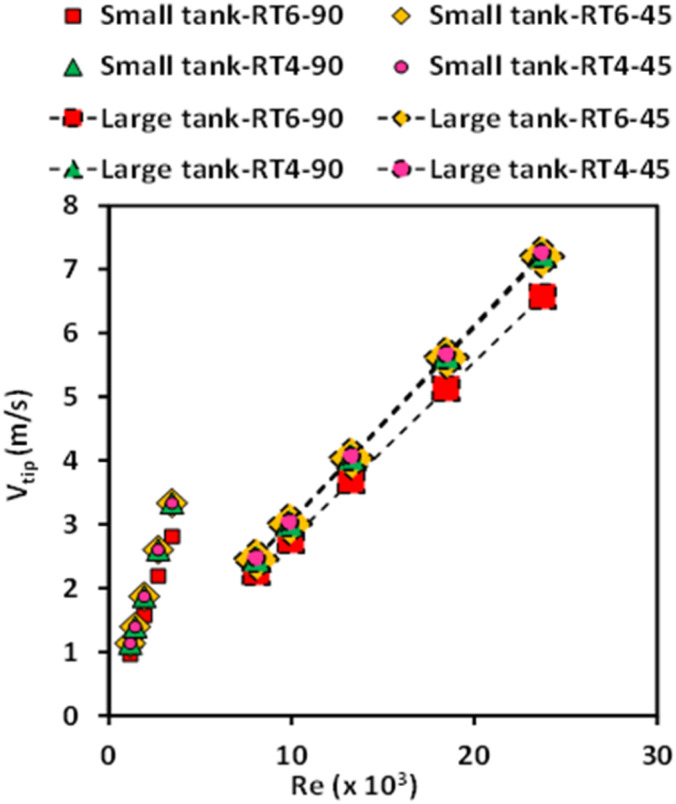
The influence of Reynolds number on the tip velocity of stirred tanks of varying sizes, equipped with different blade configurations (*i.e.*, 45° 6 blades, 90° 4 blades, 90° 6 blades, and 45° 4 blades), in the context of a Ruston turbine.

Tip velocity is a crucial parameter for designing and operating stirred tanks because it significantly influences mixing efficiency and overall process performance. Fig. 8 illustrates the relationship between the tip velocities of the smaller and larger stirred tanks and the Reynolds number. The high impeller speed causes the tip velocity to have a propensity to increase as the Reynolds number increases. The reactor scale has a notable effect on tip velocity, as an increase in stirred tank size leads to higher tip velocity due to a larger diameter of the impeller. Furthermore, the quantity and orientation of the blades exhibit a relatively minor influence on the velocity at the tip.

**Fig. 9:**
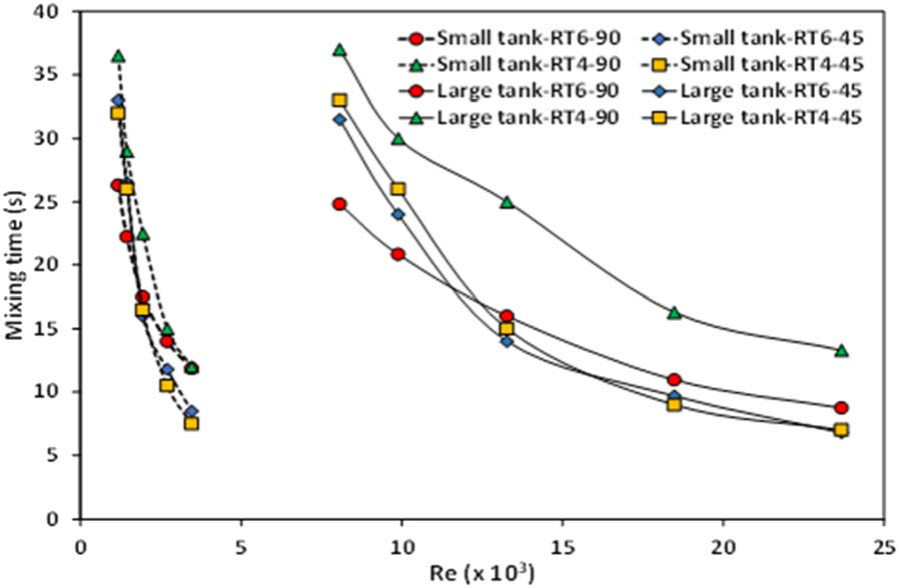
The impact of Reynolds number on the mixing time in small- and large-scale stirred vessels equipped with Ruston turbine agitators including 90° 6-blades, 45° 6-blades, 90° 4-blades, and 45° 4-blades.

The mixing time within a stirred tank is an important factor that is impacted by various factors, such as the dimensions of the tank, the design of the impeller, and the rotational speed of the impeller. The examination of mixing time in a stirring vessel can be conducted by experimental or numerical methodologies, commonly employing the tracer methodology.^53–55^ Various approaches and relationships for determining mixing time have been presented in the literature.^56^ Some researchers,^29,57^ have employed Computational Fluid Dynamics (CFD) simulations to calculate mixing time in stirred tanks. In this work, mixing time calculation based on Phumnok's proposed was utilized, and detailed information can be found in the ref. 29. Fig. 9 depicts the relationship between the mixing time seen in small and large stirred tanks and the corresponding Reynolds number. It was observed that tank size significantly influences mixing time. As the Reynolds number increases, there is a notable decrease in the mixing time observed in the small-scale stirred tank. In the context of the large agitated vessel, it was observed that the mixing time exhibited a gradual decrease as one raised the Reynolds number. The impact of impeller design, specifically the quantity and orientation of the impellers, is relatively diminished in smaller tanks.

This effect may be attributed to operating with a narrow range of Reynolds number. Conversely, in large tanks, mixing time decreases with an increasing blade number, indicating improved mixing efficiency.^44,49,50,58–61^ In addition, a 45° blade angle tends to decrease mixing time. Therefore, the optimal design is the Ruston turbine equipped with six blades set at a 45° angle.

### 3.3 Scale up of stirred tank

The process of scaling up a reactor is a pivotal undertaking in the context of commercial production, however it presents several difficulties, particularly in the case of multi-phase reactors. Various scaling laws have been proposed,^41^ and choosing the appropriate scaling law is essential for a specific application. In this study, scaling up based on a constant mixing time was monitored. The scaling law proposed by Norwood and Metzner,^62^ derived from a homogeneous liquid mixture in a single phase, was adopted as shown below,11
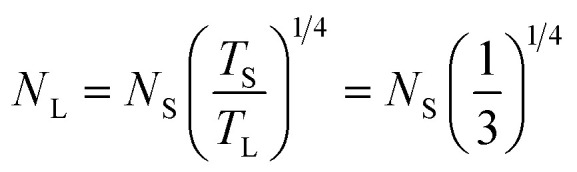


In the above equation, *N* represents the rotational speed of the impeller in revolutions per minute (rpm), whereas *T* is the diameter of the tank in meters (m). The subscripts L and S denote large-scale and small-scale reactors, respectively.

Table 2 displays the impeller speeds of the small and large stirred tanks according to the suggested scaling law.

**Table 2:** The rotational velocities of the impellers in the smaller and larger stirred tanks in accordance with the suggested scaling principle

Small tank reactor		Large tank reactor	
*N*_S_	Re (−)	*N*_L_ = 0.760*N*_S_	Re (−)
407	8247	310	55 782
500	10 142	380	68 378
671	13 621	510	91 711
934	18 950	710	127 760
1197	24 288	910	163 749

**Fig. 10:**
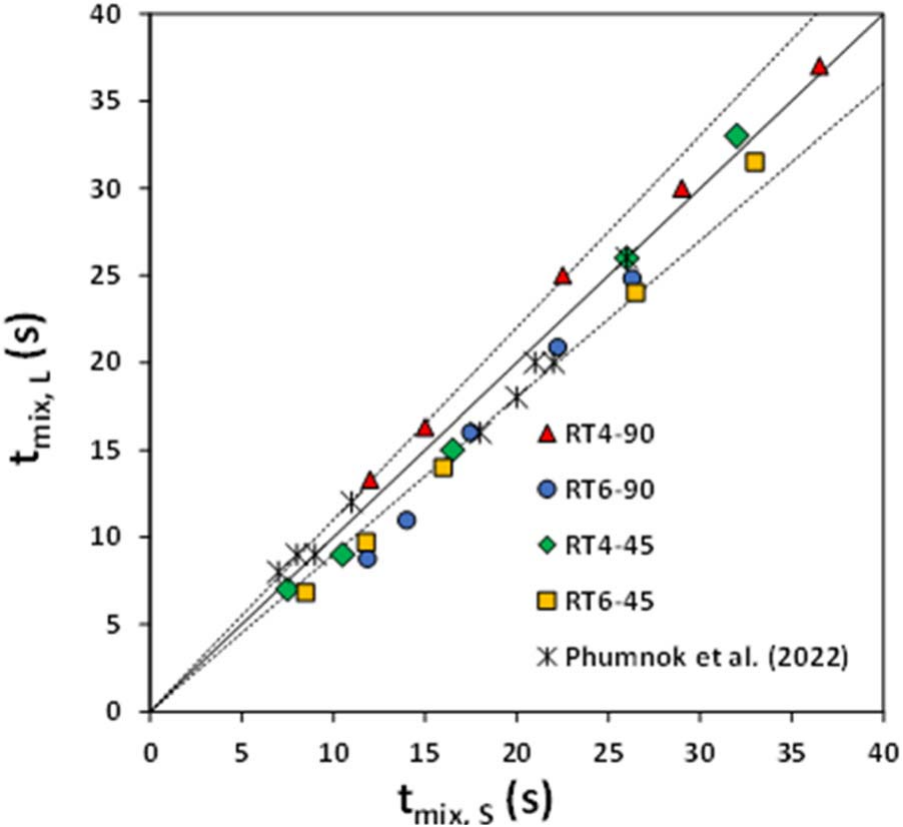
The mixing time parity plot for small and large-scale reactors equipped with 90° 6-blades, 45° 6-blades, 90° 4-blades, and 45° 4-blades Ruston turbine agitators compared with Phumnok *et al.* (2022).^29^

The effects of the number and angle of the impeller were investigated. Fig. 10 shows the parity plot of the mixing time in small- and large-scale reactors using the Norwood and Metzner upscaling law. The small and large stirred tanks have nearly identical mixing time, with a ±10% variation. Therefore, the scaling law proposed by Norwood and Metzner can be effectively used for scaling up of the Rushton turbine stirred tank for silica dispersion/natural rubber latex mixing.

## 4. Conclusion

The wet mixing method is a new technique for producing rubber composites with high silica content. Mixing efficiency is a crucial factor impacting process performance, and it is greatly influenced by impeller design, and for commercial production, upscaling this mixing technique is crucial. Flow behaviors of each fluid phase are simulated using Computational Fluid Dynamics (CFD) with a two-fluid model. Simulation results indicate that a 90° blade enhances radial flow, while a 45° blade enhances axial flow, driving most of the liquid across the impeller and increasing fluid velocity. In the blade zone, increasing the number of blades leads to a more uniform velocity distribution. Large mixing tanks exhibit higher fluid velocity. In a small tank, the impeller arrangement, specifically the number and angle of blades, has no effect on mixing time. This lack of effect may be attributed to operating within a narrow range of Reynolds numbers. In contrast, it has been shown that larger tanks see a reduction in mixing time as the number of blades rises. Furthermore, a 45° blade angle tends to decrease mixing time. Therefore, the optimal design is a Ruston turbine with six blades set at a 45° angle. The study utilizes the scale-up criterion suggested by Norwood and Metzner, demonstrating the reliable scaling of natural latex containing high silica content within a range of ±10%.

## Abbreviations

*C*
: Clearance of the impeller plane off the bottom of the stirred tank (m)
*C*_D_
: Drag coefficient
*D*_L_
: Diameter of the impeller of the large stirred tank (m)
*D*_S_
: Diameter of the impeller of the small stirred tank (m)
*F*
: Interphase force (N)
*F*_drag_
: Drag force (N)
*H*
: Height of the liquid in the stirred tank (m)
*H*_L_
: Height of the liquid in the large stirred tank (m)
*H*_S_
: Height of the liquid in the small stirred tank (m)
*k*
: Turbulent kinetic energy (m^−2^ s^−2^)
*K*
: Proportional factor
*n*
: The total number of the data
*N*
: The speed of the agitator (rpm)
*N*_L_
: The speed of the agitator of the large stirred tank (rpm)
*N*_S_
: The speed of the agitator of the small stirred tank (rpm)
*P*
: Pressure (Pa)
*R*
: The radius of the agitator (m)
Re_d_: Particle Reynolds number
*r*
: Radial coordinate (m)
*S*
: Source term
S.D.: The standard deviation
*t*
: Time (s)
*t*_1_
: Mean eddy lifetime (s)
*t*_p_
: Particle response time (s)
*t*_95_
: 95% of the perfect uniformity (s)
*t*_mix_
: The mixing time (s)
*T*
: Diameter of the stirred tank (m)
*T*_L_
: Diameter of large stirred tank (m)
*T*_S_
: Diameter of small stirred tank (m)
*u*
: Mean velocity component (m s^−1^)
*u*′: Fluctuation of the velocity component (m s^−1^)
**u**
: Velocity vector (m s^−1^)
*v*_*kt*_
: The kinematic turbulent viscosity of phase *k*
*V*_tip_
: The tip velocity (m s^−1^)
*x*_*i*_
: The volume fraction at position *i*
*x̄*
: The average volume fraction
*z*
: Axial coordinate starting from the tank bottom (m)

### Greek symbols

α
: Holdup
*δ*_*ij*_
: Kronecker delta
ε
: Turbulent kinetic energy dissipation rate (m^2^ s^−3^)
μ
: Viscosity (Pa s)
ρ
: Density (kg m^−3^)
σ
: Interfacial tension (N m^−1^)
*σ*_t_
: Schmidt number
ϕ
: General variable

### Subscripts

c: Continuous phase
d: Dispersed phase
drag: Drag force
*i*, *j*: *i* and *j* directions
*k*
: *k* th phase
lam: Laminar
t: Turbulence

## Conflicts of interest

There are no conflicts to declare.

## Supplementary Material
